# Supplementary material for: A mechanosensory receptor required for food texture detection in Drosophila
Source: Nat Commun. 2017 Jan 27;8:14192. doi: 10.1038/ncomms14192 (PMC5290141; doi:10.1038/ncomms14192)
Supplement: Supplementary Information — Supplementary Figures. [file ncomms14192-s1.pdf]

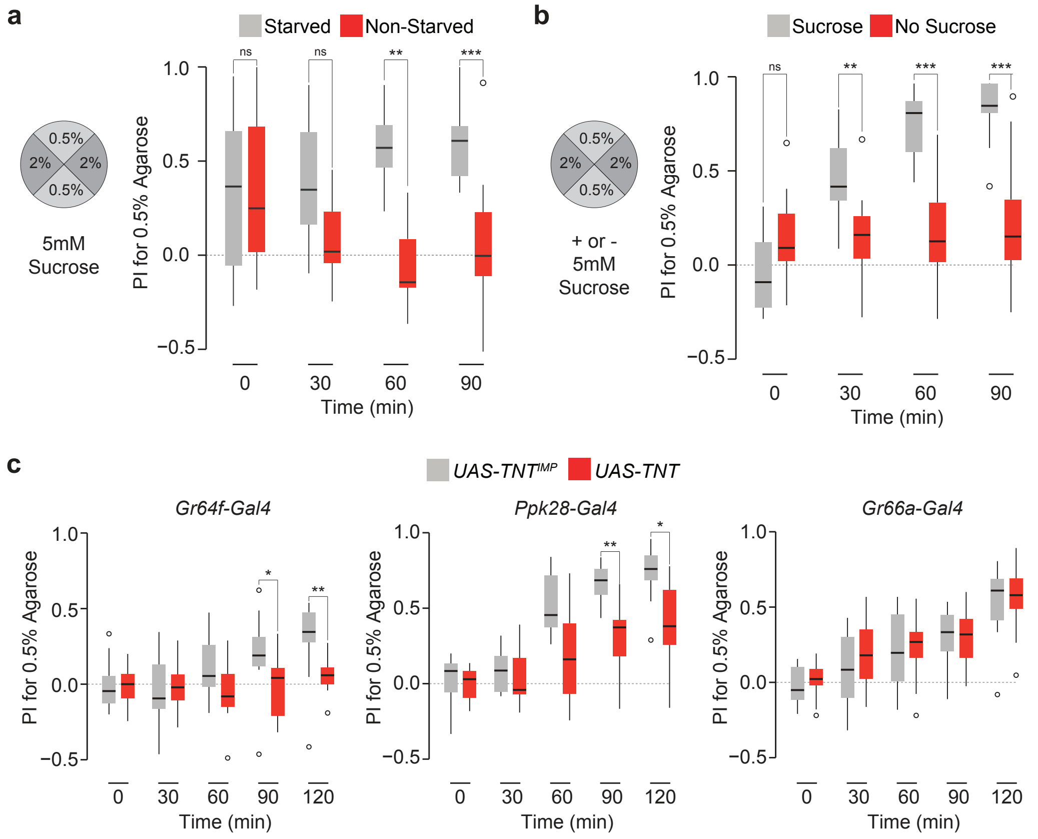

Supplementary Figure 1 | Influence of the absence of external gustatory stimuli and gustatory pathway inhibition on texture discrimination behaviour.

(**a**) Time course of preference indices (PI) of starved (grey) or non-starved (red) wild-type flies for 0.5% agarose in a 0.5% versus 2% arena with 5 mM sucrose (n=12 for each conditions). ns: not significant, *** P<0.001, ** P<0.01 (Wilcoxon rank sum test). The 90 min time-point corresponds to the data shown in Fig. 2a.

(**b**) Time course of PI of wild-type flies for 0.5% agarose in a 0.5% versus 2% arena assay in the presence (grey) or absence (red) of 5 mM sucrose (n=13 with sucrose, n=14 without sucrose). ns: not significant, *** P<0.001, ** P<0.01 (Wilcoxon rank sum test). The 90 min time-point corresponds to the data shown in Fig. 2b (left).

(**c**) Time course of PI for 0.5% agarose in a 0.5% versus 2% arena assay (with 5 mM sucrose) of flies in which different gustatory sensory neuron populations are inhibited through transgenic expression of Tetanus toxin (TNT) under the control of *Gr64f*, *Ppk28* or *Gr66a* promoters at four different time-points (the 120 min time-points correspond to the data shown in Fig. 2d; see Fig. 2d legend for genotypes). Control *Gr64f-Gal4/UAS-TNT*^{IMP} animals have delayed preference for 0.5% agarose, but are statistically distinct from *Gr64f-Gal4/UAS-TNT* animals at both 90 and 120 min. ** P<0.01, * P<0.05 (Wilcoxon rank sum test). Only significant differences are indicated.

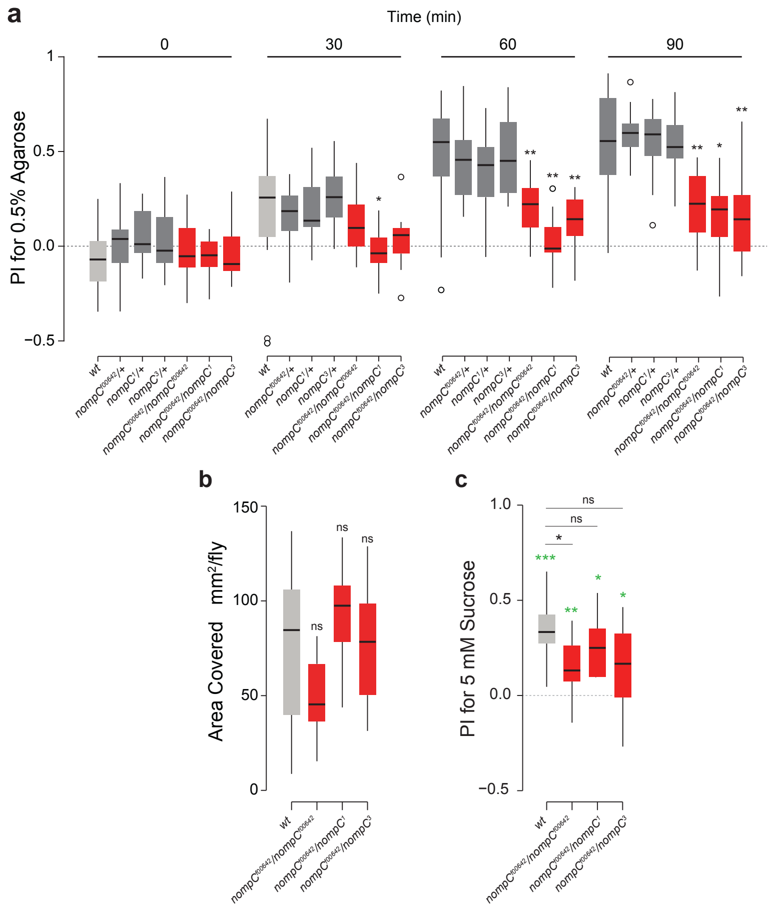

Supplementary Figure 2 | *nompC* mutants exhibit reduced texture preference but normal exploratory and chemosensory preference behaviour.

(**a**) Time course of the PI of the indicated control and *nompC* mutant flies for 0.5% agarose in a 0.5% versus 2% arena assay (with 5 mM sucrose). Wild-type (n=17), *nompC*^{f00642}/+ (n=18), *nompC*^{1}/+ (n=15), *nompC*^{3}/+ (n=15), *nompC*^{f00642}/*nompC*^{f00642} (n=17), *nompC*^{f00642}/*nompC*^{1} (n=10), *nompC*^{f00642}/*nompC*^{3} (n=12). All comparisons were made against the wild-type control (Wilcoxon rank sum test with Bonferroni correction for multiple comparisons). Only significant differences are indicated; ** P<0.01, * P<0.05. The 90 min time-point is shown in Fig. 3a.

(**b**) Exploratory behaviour by the indicated control and *nompC* mutant flies of an arena containing 0.5% versus 2% agarose with 5 mM sucrose. *Wild-type* (n=24), *nompC*^{f00642}/*nompC*^{f00642} (n=12), *nompC*^{f00642}/*nompC*^{1} (n=13), *nompC*^{f00642}/*nompC*^{3} (n=9). ns: not significant. (Wilcoxon rank sum test with Bonferroni correction for multiple comparisons).

(**c**) PI of the indicated control and *nompC* mutant flies for 5 mM sucrose in an arena assay with 0 mM versus 5 mM sucrose quadrants in a uniform concentration of 0.5% agarose. Wild-type (n=13), *nompC*^{f00642}/*nompC*^{f00642} (n=12), *nompC*^{f00642}/*nompC*^{1} (n=5), *nompC*^{f00642}/*nompC*^{3} (n=15). ns: not significant, ** P<0.01, * P<0.05 (Wilcoxon rank sum test with Bonferroni correction for multiple comparisons; green asterisks indicate Wilcoxon signed rank test (H_0=0)).

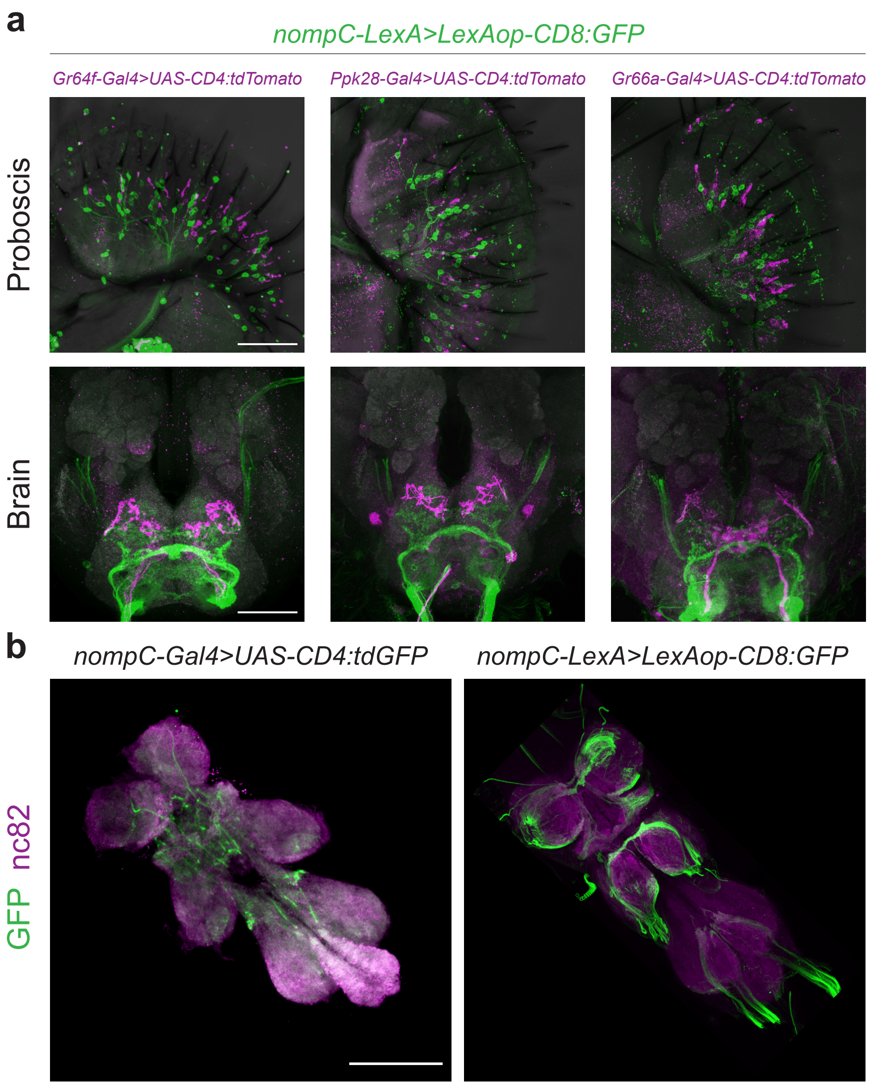

Supplementary Figure 3 | Anatomical comparison of neuron populations expressing drivers for *nompC* and gustatory sensory neuron classes.
(**a**) Immunofluorescence with anti-GFP (green) and anti-RFP (magenta) on whole-mount proboscides (top) and brains (bottom) of animals expressing membrane-targeted GFP and Tomato reporters under the control of the indicated combinations of promoters. The grey background represents the bright-field image of the proboscides, and immunofluorescence signal with the synaptic neuropil marker nc82 in the brains. Arrowheads point to areas of overlap between GFP and Tomato signals. Genotypes:
w;LexAop-CD8:GFP-2A-CD8:GFP/Gr64f-Gal4;nompC-LexA/UAS-CD4:tdTomato
w;LexAop-CD8:GFP-2A-CD8:GFP/Ppk28-Gal4;nompC-LexA/UAS-CD4:tdTomato
w;LexAop-CD8:GFP-2A-CD8:GFP/Gr66a-Gal4;nompC-LexA/UAS-CD4:tdTomato
Scale bars, 50 μm.
(**b**) Immunofluorescence with anti-GFP (green) and nc82 (magenta) on whole-mount ventral nerve cords of animals expressing a membrane-targeted GFP reporter under the control of the indicated *nompC*-promoter drivers. Genotypes:
w;UAS-CD4:tdGFP;nompC-Gal4 (left)
w;LexAop-CD8:GFP-2A-CD8:GFP;nompC-LexA (right).
Scale bar, 50 μm.
